# Editorial: New insights into the pathogenesis of idiopathic inflammatory myopathy

**DOI:** 10.3389/fimmu.2025.1639383

**Published:** 2025-06-27

**Authors:** Dana P. Ascherman

**Affiliations:** Department of Medicine, Division of Rheumatology and Clinical Immunology, University of Pittsburgh School of Medicine, Pittsburgh, PA, United States

**Keywords:** myositis, pathogenesis, interferon, RNA sequencing, autoantibody

The idiopathic inflammatory myopathies (IIM) represent a group of systemic autoimmune disorders in which muscle is inappropriately targeted for immune-mediated destruction. Extra-muscular complications may involve the skin, joints, vasculature, and lungs, with significant impact on morbidity and mortality. Current therapy is effective, but requires the use of potent immunosuppressive agents that are relatively non-specific and carry great risk of side effects such as infection. Based on these considerations and gaps in our understanding of disease pathogenesis, there is a clear need for expanded research and new disease models to facilitate development of more targeted therapy in this potentially devastating disease process.

Unfortunately, there is an extreme paucity of *in vitro* models capable of replicating many of the key cellular interactions that ultimately dictate immune infiltration of target tissues such as muscle and lung. As a result, previous investigation has focused on the development of *in vivo* animal models that are either genetically driven or antigen-induced. While these models capture some of the clinical and immunological features of human disease, most do not recapitulate extra-muscular manifestations such as interstitial lung disease. However, with recent advances in multi-omics technology, we now have the tools to better interrogate and define relevant disease pathways in existing disease models as well as human tissues—which should facilitate the development of novel therapeutic targets.

Over the last several years, application of these newer tools and technologies have begun coming to fruition. Advancements include the characterization of K_2p_2.1, a potassium channel that may regulate influx of inflammatory cells into diseased muscle tissue (1), and provocative studies demonstrating the ability of autoantibodies to penetrate cells and inhibit key cellular processes impacting transcriptional regulation and downstream signaling pathways (2). In this special section focusing on the pathogenesis of IIM, the brief compendium of manuscripts highlights additional avenues of discovery that have advanced our understanding of aberrant immune responses and metabolic profiles characterizing different disease subtypes. For example, Wang et al. present data demonstrating the ability of various byproducts of glycerophospholipid metabolism and fatty acid oxidation to distinguish different antibody subgroups of dermatomyositis, high versus low disease activity, and the presence versus absence of interstitial lung disease. Illustrating the complementary application of single cell RNA sequencing of peripheral blood mononuclear cells (PBMC), the analyses presented by Ding et al. provide compelling insight to differences in immune landscape (cellular profile, cytokine signaling pathways) and metabolic derangements between patients with anti-synthetase antibody-positive dermatomyositis and anti-MDA5 antibody-positive dermatomyositis. In a different approach, Bolko et al. use single molecule array (SIMOA) and ELISA to characterize Type I interferon profiles in different autoantibody subsets of dermatomyositis—with results demonstrating subset-dependent correlations of IFNα and/or IFNβ with disease activity. Finally, Gao et al. examine clinical, immunological, and demographic risk factors associated with risk of Pneumocystis jirovecii infection (documented by metagenomic sequencing) and poor outcome in MDA5 antibody-positive dermatomyositis. Collectively, these studies demonstrate the power of different omics approaches in elucidating pathogenically-relevant pathways in IIM and offer hope of defining newer, desperately needed therapeutic targets.
